# A New Theory and Treatment of Disease, Founded on Natural Principles

**Published:** 1843-04

**Authors:** 


Art. VIII.—*A New Theory and Treatment of Disease, founded on Natural Principles.* By John Tinnion, m.d. Ayr.—*Edinburgh*, 1843. 8vo, pp. 35.

At last, we verily believe, folly and absurdity in medical literature have reached their acmè, in the production now before us. If Ayr really and truly claims the author of this pamphlet among its citizens, then Ayr, we hesitate not to say, can boast of an unparalleled blockhead; and the college which dubbed John Tinnion an M.D., deserves to be disfranchised. But the thing is altogether so wretched that we are disposed to believe that the whole is a hoax got up by some wag to ridicule the profession. If so, the attempt must fail, from the unmitigated dulness of a joke that drags its slow length along three dozen mortal pages.

